# Instruments for assessing social support in social networks and in the self-management and rehabilitation process of persons poststroke: a scoping review protocol

**DOI:** 10.1136/bmjopen-2025-106975

**Published:** 2025-10-15

**Authors:** Marcus Falk Johansson, Afsaneh Taei, Linnea McCarthy, Catharina Gustavsson, Signe Tomsone, Maya Kylén, Marie Elf

**Affiliations:** 1 School of Health and Welfare, Dalarna University, Falun, Sweden; 2 Center for Clinical Research Dalarna, Uppsala University, Falun, Sweden; 3 Department of Public Health and Caring Sciences, Uppsala University, Uppsala, Sweden; 4 Departament of Rehabilitation, Riga Stradins University, Riga, Latvia; 5 Faculty of Health Science, Kristianstad University, Kristianstad, Sweden; 6 Department of Health Sciences, Lund University, Lund, Sweden

**Keywords:** Stroke, Social Support, Rehabilitation medicine

## Abstract

**Abstract:**

**Introduction:**

As care and rehabilitation poststroke are increasingly moving into persons’ home environment, the importance of support from social networks in self-management and rehabilitation has emerged as an important topic for research and practice. While there are instruments used to assess social support and collective efficacy, a clearer scope of the availability and quality of these instruments is needed. This clarification will enable the development of interventions integrating social network perspectives in poststroke rehabilitation.

**Methods and analysis:**

To assess the availability and quality of instruments assessing social support and collective efficacy, a scoping review will be conducted and reported following the Preferred Reporting Items for Systematic Reviews and Meta-analyses Extension for Scoping Reviews guidelines (PRISMA-ScR). Literature searches conducted between 14 November 2024 and 15 November 2024 in the CINAHL and PubMed/Medline databases resulted in 4631 articles potentially eligible. After removing duplicates, 4121 articles’ titles and abstracts were initially screened. Full-text screening, searches of reference lists and data extraction started in June 2025. Starting August 2025, two reviewers will assess the full texts against the inclusion criteria in Covidence using a coding template. Identified instruments will be appraised following the COSMIN (Consensus-based Standards for the selection of health Measurement INstruments guidelines) and analysed using a narrative descriptive method. Results will be reported in February 2026 according to PRISMA-ScR guidelines.

**Ethics and dissemination:**

Ethical approval is not required for this scoping review, as it does not involve primary data. However, this review follows established ethical guidelines and best practices, and included studies will be reviewed to ensure that they received ethical approval and included informed consent. Results from the review will be disseminated through an article in a scientific journal, at relevant conferences and surmised to stroke organisations. A policy brief will be developed for health and social care professionals and policy makers.

**STRENGTHS AND LIMITATIONS OF THIS STUDY**

- The review will follow the PRISMA-ScR guidelines, which strengthens methodological transparency and reporting.
- Two reviewers will independently screen and extract data, reducing the risk of bias.
- Instruments will be appraised with reference to the COSMIN (Consensus-based Standards for the selection of health Measurement INstruments guidelines) framework to identify reported validation aspects.
- Only studies including adults aged 18 years or older will be included, which may limit insight into younger populations.
- Only English-language articles will be included, which may lead to language bias.

## Introduction

 As stroke care increasingly shifts toward home-based rehabilitation and shorter hospital stays, the importance of self-management support has grown.^1^,^3^ This transition places greater responsibility on stroke survivors and their families to manage not only their health but also their daily activities, participation in society and emotional well-being after discharge.^4 5^ For many, this is particularly challenging as stroke is commonly associated with physical disabilities, fatigue, cognitive impairments and changes in social roles.^6^,^8^

The growing expectations placed on individuals become especially concerning given the widespread prevalence and impact of stroke worldwide. Globally, stroke remains the third-leading cause of disability worldwide,^9^ with over 15 million people experiencing a stroke annually.^10^ In Sweden, approximately 25 000 persons experience a stroke each year, the majority being older adults with an average age of 72 years at onset.^11^ For many in this population, managing rehabilitation independently is a significant challenge. Studies report a range of unmet needs and lack of sufficient support, particularly during the transition from hospital to home.^12^ This feeling of being lost after discharge may stem from existing health services that do not adequately support and address stroke survivors’ complex, individual needs.^12^ Social support is often lacking,^5 12 13^ yet access to social support is a known predictor of recovery outcomes.^4^ Social support is a multifaceted concept, with predominantly four different attributes: emotional, instrumental, informational and appraisal support^14^ a person receives from others with whom they have positive ties, commonly called a social network.^15 16^ While social networks could refer to social media platforms, this study uses the term to describe the environment of social relations a person has, such as family, friends, peers or community.

Social support can significantly impact an individual’s ability to cope with the consequences of long-term conditions and to actively engage in effective self-management. With direct impact on outcomes such as mental health, functional independence and reintegration into daily life. It has been shown to help individuals manage emotional and practical challenges, return to work and enhance quality of life poststroke.^17 18^ Vassilev and colleagues^19^ found that a person’s social network can contribute to the self-management process, for instance, by enabling knowledge-sharing, modelling of behaviours, facilitation of motivation and influencing lifestyle changes. Developing self-management skills has been shown to improve health outcomes, enhance psychological well-being and support greater independence in daily life.^120^,^23^ By fostering confidence, problem-solving abilities and a sense of control, self-management approaches may help stroke survivors better navigate the complexities of recovery and community reintegration.

Traditionally, self-management has been conceptualised as an individual skill, theoretically based on self-determination theory and the concept of self-efficacy of social cognitive theory.^24^,^26^ However, this perspective has evolved and today, self-management is increasingly understood as a collective process involving the individual’s social networks, such as family and friends, professionals and others.^26 27^

Bandura^25^ defines collective efficacy as ‘a group’s shared belief in its ability to organize and execute the actions required to achieve certain goals’. This concept has been applied in rehabilitation, among vulnerable populations, and within educational settings.^26 28^ Extending the concept of self-efficacy to a collective process makes it particularly relevant for stroke rehabilitation, where survivors are part of a social network that can mobilise resources, provide support and coordinate efforts to help them face the challenges of recovery.^29 30^

As more people survive strokes and return to life in the community, the health and social care systems face growing challenges in supporting their complex and long-term care needs.^31^ Many stroke survivors report unmet support needs in re-establishing their social networks and roles, which are critical for recovery, well-being and community participation.^32 33^ Given the increasing reliance on informal care networks in long-term care, including poststroke,^34 35^ attention has turned to the role of social networks and the capacity they represent.

Although there is growing interest in the social dimensions of stroke rehabilitation, and several studies have employed instruments to assess social networks and related concepts, no comprehensive overview or mapping of these instruments currently exists. It remains unclear which instruments are available, how they have been used with stroke populations and whether they have provided evidence of validity, reliability and suitability for rehabilitation settings.

An overview of existing instruments can provide health- and social care professionals with knowledge about the range of available tools, how they have been used to assess social support of people who have experienced a stroke and the evidence of their validity and reliability. Such a tool may help reveal areas where targeted interventions are needed to strengthen social support and thus improve rehabilitation outcomes.

While measurements on social support and collective efficacy instruments have been developed for other long-term conditions such as diabetes, their suitability for stroke remains largely underexplored. Stroke survivors often face cognitive and functional challenges that may affect how they interact with their networks. Therefore, care providers need strategies to support people poststroke and use their social networks as resources for day-to-day management and support.

Understanding the scope and quality of instruments available to assess social support and collective efficacy may provide a stepping stone to develop interventions integrating social network perspectives in stroke rehabilitation.

### Study objective

This scoping review will map the scope and quality of instruments used to assess social support from social networks and collective efficacy in rehabilitation for adults aged 18 or above who have experienced a stroke to provide an overview of available instruments. The scoping review will include instruments designed for stroke and other long-term conditions and map out the use and applicability in a stroke rehabilitation context. The overall research question is: What instruments are available to measure social support and collective efficacy in the rehabilitation of adults living with stroke?

### Three specific study objectives will structure the review

1. To identify instruments that measure social support and collective efficacy, used in rehabilitation of people with long-term conditions or stroke.
2. To examine the characteristics of these instruments, including their objectives and scope, structure, theoretical foundations and the settings in which they have been used.
3. To identify and summarise the existing evidence regarding the methodological quality, reliability and validity of each identified instrument and its use in research and clinical practice in the rehabilitation of people with stroke.

## Methods

The scoping review follows the widely recognised methodological framework established by Arksey and O’Malley,^36^ which is well-suited to our aim of identifying, categorising and analysing instruments used to assess social support from social networks and collective efficacy. In addition to mapping available tools, we will examine their reported validity, reliability and applicability across populations and settings. This level of analysis aligns with refinements proposed by Levac *et al*^37^ and the guidance from the Joanna Briggs Institute,^38^ which emphasise the importance of analytical depth in the synthesis stage. The protocol is reported in accordance with the Preferred Reporting Items for Systematic Reviews and Meta-analyses Extension for Scoping Reviews (PRISMA-ScR).^39^ The protocol is registered with the Open Science Framework^40^ (Doi. 10.17605/OSF.IO/JYMWR).

### Development of the search strategy

Database searches were conducted in Ovid MEDLINE/PubMed on 14 November 2024, and in CINAHL and Embase on 15 November 2024. The search strategy was developed based on recommendations for conducting scoping reviews and expert advice from a research librarian. The PEO (Population, Exposure, Outcome) framework^41^ was used to clarify and focus the review, defining the population, exposure and outcomes and structure search blocks. Population (P): The population block includes adults (18 years old or older),^1^ who had a stroke or have long-term conditions. This block also encompasses contexts related to stroke, rehabilitation and long-term conditions. Exposure (E): The exposure block focuses on social factors, specifically social networks, social support and collective efficacy. These are the specific elements or variables that are being examined in relation to the population. Outcome (O): The outcome block involves the instruments, scales and measurements used to assess the factors mentioned in the exposure block. These instruments evaluate the quality, validity and suitability of social support and collective efficacy.

The refining and development of the search strategy was iterative, with pilot searches conducted in CINAHL, Embase and Ovid MEDLINE/PubMed. These pilots informed a key decision not to limit the population to individuals with stroke, as it may risk excluding instruments commonly used across various long-term conditions with similar social networks and collective efficacy assessment needs. Moreover, we considered that instruments used in the context of long-term conditions, such as cardiovascular disease or diabetes, may also be suitable for stroke populations. A detailed search strategy was developed for MEDLINE/PubMed and adapted for CINAHL and Embase. The full search strategy for MEDLINE/PubMed is presented in table 1.

**Table 1:** Search strategy for MEDLINE/PubMed, conducted 14 November 2024

#	Search	Hits
1	exp Psychometrics/	93 442
2	exp “Surveys and Questionnaires”/	1 274 975
3	(measur* or instrument? or indicies or index* or inventory or inventories or scale? or screen* or surve* or checklist* or questionnaire* or tool? or observation form? or tally sheet? or sociometric device*).ti.	1 281 150
4	or/1–3	2 293 337
5	exp Collective Efficacy/	16
6	exp Social Networking/	6483
7	exp Social Support/	83 223
8	collective efficac*.ti.	207
9	social network*.ti.	7062
10	(support* adj3 (social* or peer? or group?)).ti.	18 162
11	or/5–10	97 510
12	exp Stroke/	186 239
13	exp Stroke Rehabilitation/	18 904
14	stroke.ti,ab,kf.	339 579
15	((chronic* or long term) adj3 (ill or illness* or disease? or condition? or manag* or recover*)).ti,ab,kf.	521 692
16	or/12–15	890 085
17	4 and 11 and 16	1611

To create an effective search string, Boolean operators and truncations were used with synonyms or related items connected to each block with OR operator to capture variations. The AND operator was used between blocks to narrow the search to studies that mentioned terms from key areas.

The literature search was conducted in CINAHL, Embase and MEDLINE/PubMed databases. A combination of Medical Subject Heading terms and keyword searches were used to identify publications that meet inclusion criteria. Different databases handle Boolean operators, truncation and field-specific searches differently.

In addition, the reference list of each included study will be searched to identify further studies. Finally, instruments identified as relevant for the study population will be used as specific search terms.

### Eligibility criteria

Eligible studies were selected based on the following inclusion and exclusion criteria. Studies were included if they:

- Reported on the development, validation or use of instruments that measure social support, social networks and/or collective efficacy.
- Included adult populations.
- Were quantitative empirical studies.
- Were published in English.
- Were published from 1 January 2000.

Studies were excluded if they:

- Did not include an instrument or tool that measures either social support, social networks or collective efficacy.
- Focused exclusively on theoretical models without application or development of measurement tools.
- Were editorials, opinion pieces or conference abstracts.

### Screening procedure

After removing duplicates, title and title and abstract screening, references were exported to Covidence (www.covidence.org). During title and abstract screening, the research team discussed a representative sample of studies to ensure consistency between their interpretation of social networks and collective efficacy. To further test the inclusion criteria, one of the researchers (AT) screened the abstracts of (n=128) selected articles to determine whether they should proceed to full-text screening. Following this, two other researchers (ME, MFJ) conducted a blinded pilot screening of titles and abstracts for 20% of these articles (n=26) before the full blinded abstract screening was carried out. Studies that meet the inclusion criteria were retrieved in full text. Starting August 2025, two reviewers (MFJ, AT) will assess the full texts against the inclusion criteria. A meeting will be held to verify a random selection of each reviewer’s studies and discuss any studies reviewers judge uncertainly, after which group decisions will be made to exclude or include them. Finally, each article’s reference list will be searched to identify further studies. According to the scoping review method guide, we will not perform a quality appraisal of the articles. Instead, the quality of the instruments will be reviewed according to the Consensus-based Standards for the selection of health Measurement INstruments guidelines (COSMIN).^42^ This includes identifying measures of reliability and validity (content validity, construct validity and criterion validity), sensitivity and responsiveness of each instrument.

### Charting the data

Two reviewers (MFJ, AT) will conduct independent data extraction using Covidence (www.covidence.org), a web-based collaboration software platform that streamlines the production of systematic and other literature reviews. Information about the study, that is, authors and country, the setting, long-term conditions and the tool used will be extracted. Data extracted about the tools will also be extracted, that is, its purpose and scope, target group definitions of social support, social network and collective efficacy, theoretical basis, development method, number of items and domains. A summary table will chart each instrument’s rating according to COSMIN across relevant measurement properties. This will provide an overview of methodological quality across instruments.

### Compile, summarise and report the results

The extracted data will be summarised and analysed using a narrative descriptive method^43^ providing a structured synthesis of each instrument’s strengths and limitations, informed by COSMIN’s assessment criteria. The results will be reported according to PRISMA-ScR guidelines.^39^ Summary tables will present key information for each instrument, including COSMIN classifications across all relevant measurement properties, to enable easy comparison and support the selection of high-quality instruments for clinical and research purposes.

### Patient and public involvement statement

Persons with stroke will not be directly involved in conducting this scoping review. However, a reference group including individuals with lived experience, family members and professionals contributed to discussions prior to the project and will be consulted when interpreting and disseminating the findings.

### Ethics and dissemination

This scoping review did not require ethical approval as it does not involve human participants or personal data. However, the included studies will be reviewed for ethical approval, informed consent and protection of participants’ privacy. The included studies should report that they have obtained ethical approval from relevant committees. Potential ethical issues and biases identified during the review process will be addressed in the analysis. This review follows established ethical guidelines and best practices.

For further information regarding ethical review in Sweden, please contact the Swedish Ethical Review Authority (https://etikprovningsmyndigheten.se/en/).

Results from this scoping review will be disseminated through publication in a scientific journal and at relevant conferences. Finding will also be shared among national stroke organisations and summarised into guidelines for health and social care professionals and policymakers to make results accessible and useful in clinical applications.
