# Supplementary material for: Instruments for assessing social support in social networks and in the self-management and rehabilitation process of persons poststroke: a scoping review protocol
Source: BMJ Open. 2025 Oct 15;15(10):e106975. doi: 10.1136/bmjopen-2025-106975 (PMC12530430; doi:10.1136/bmjopen-2025-106975)
Supplement: Supplementary file 1 [file bmjopen-2025-106975.reviewer_comments.pdf]

PEER REVIEW HISTORY

BMJ Open publishes all reviews undertaken for accepted manuscripts. Reviewers are asked to complete a checklist review form and are provided with free text boxes to elaborate on their assessment. These free text comments are reproduced below.

ARTICLE DETAILS

Title (Provisional)

Instruments for Assessing Social Support in Social Networks in the Self-Management and Rehabilitation Process of Persons Post-Stroke: A Scoping Review Protocol

Authors

Falk Johansson, Marcus; Taei, Afsaneh; McCarthy, Linnea; Gustavsson, Catharina; Kylén, Maya; Tomsone, Signe; Elf, Marie

---

VERSION 1 - REVIEW

**Reviewer**	**1**
**Name**	**Thayabaranathan, Tharshanah**
**Affiliation**	**Monash University, Department of Medicine**
**Date**	**25-Aug-2025**
**COI**	**None**

Thank you for the opportunity to review this manuscript entitled "Instruments for Assessing Social Support in Social Networks in the Self-Management and Rehabilitation Process of Persons Post-Stroke: A Scoping Review Protocol." I appreciate the effort in developing this work; however, there are a number of points that I think require clarification and strengthening:

Overall aim

I found it difficult to clearly understand the aim of the paper. At present, the purpose of conducting a scoping review is not well justified. If validated instruments already exist, the rationale for a scoping review is unclear. If the intent is to assess efficacy, that would usually require a systematic review rather than a scoping review. This lack of clarity makes it hard to follow the overall direction and contribution of the paper.

I recommend revisiting the aims and clarifying: What precise question the review is trying to answer; Why a scoping review is the most appropriate design (and not a systematic review or meta-analysis); How the outcomes will advance knowledge or practice in this field.

Terminology and clarity

The term “social networks” is often associated with social media, which may cause confusion. It may be more accurate to use consistent terminology such as “social support,” “social circle,” or “informal support persons” throughout the manuscript. At present, terms are used interchangeably (e.g., social networks, supportive networks, informal support), which reduces transparency and clarity.

Population criteria

The decision to restrict the population to adults aged 40 years and above is not well justified. Why exclude younger adults (18–39 years)? Please provide a rationale for this age cutoff.

Introduction

The introduction is somewhat jumbled and would benefit from re-structuring for clarity and logical flow.

A reference is required for the statement on lines 12–14 of page 5.

Instruments

The manuscript refers to “available instruments” but does not provide examples. Please clarify which instruments you are referring to.

Are you referring to quality of life measures (which are already validated and for which efficacy is known), or other tools? Please specify.

Overall, the manuscript would benefit from a clearer distinction between the intended purpose of a scoping review versus a systematic review, and from more precise and consistent use of terminology. These revisions will help strengthen the focus and transparency of the work.

VERSION 1 - AUTHOR RESPONSE

**Reviewer’s comment**	**Author’s response**
Overall aim – I found it difficult to clearly understand the aim of the paper. At present, the purpose of conducting a scoping review is not well justified. If validated instruments already exist, the rationale for a scoping review is unclear. If the intent is to assess efficacy, that would usually require a systematic review.	Thank you for this comment. We have refined the overall aim to make it clearer that the purpose of the scoping review is to map and summarize the existing instruments for assessing social networks and collective efficacy in stroke rehabilitation.<br><br>For more clarification, we have revised the *third study objective* and mentioned that the review will identify and summarize evidence regarding the reliability, validity, and use of each identified instrument. This ensures that the aim aligns with a scoping review methodology, by focusing on identification and mapping rather than evaluating intervention efficacy.

The term "social networks" may cause confusion with social media. Terms are used interchangeably, which reduces clarity.	We disagree with this notion; "social networks" is an established term in research on social support and collective efficacy. However, on p. 3 we have clarified that the term does not relate to social media and specified that we refer to social networks as the environment of social relations a person has (p.3.).<br><br>In the remainder of the text, social networks will be used consistently.
Why restrict the population to adults aged 40 years and above? Why exclude younger adults?	Thank you for raising this point. In the abstract and aim it was stated that the age limit was set to 40 years old or above, this was an error and, in the methods section, it is stated that the age limit is 18 years old or older. We have corrected this mistake.
The introduction is somewhat jumbled and would benefit from re-structuring for clarity and logical flow.	We agree and have revised the introduction to hopefully improve its structure and logical flow.
Reference required (page 5, lines 12–14).	When restructuring the introduction, this section was merged with other text, and references were added to support our statement (reference, 25-26).
The manuscript refers to "available instruments" but does not provide examples. Are you referring to quality of life measures or other tools?	As the overarching research question concerns to map and provide an overview of available instruments that measure social support and collective efficacy specifically. We believe that it would not be fruitful to provide examples of the instruments we aim to map.
The manuscript would benefit from clearer distinction between scoping and systematic review, and more precise terminology.	We have revised the manuscript to emphasize that the aim is to provide a **mapping and overview** (appropriate for a scoping review), not to evaluate efficacy. Terminology has been clarified and made consistent throughout.
